# Sirolimus-induced bronchiolitis obliterans organizing pneumonia in a kidney transplant recipient; a case report and review of literature

**DOI:** 10.12860/jnp.2014.21

**Published:** 2014-07-01

**Authors:** Behzad Einollahi, Jafar Aslani, Mehrdad Taghipour, Mohsen Motalebi, Hamidreza Karimi-Sari

**Affiliations:** Nephrology and Urology Research Center, Baqiyatallah University of Medical Sciences, Tehran, Iran

**Keywords:** Pneumonia, Sirolimus, Transplant

## Abstract

*Background:* Sirolimus is immunosuppressive drug used to prevent rejection in kidney transplantation. Pulmonary problems are one of the serious complications which may be seen after administration of this drug and it is believed that it could be life threatening.

*Case Presentation:* Here in this paper we presented a 49-years-old man with bronchiolitis obliterans organizing pneumonia (BOOP) induced by chronic use of Sirolimus. The disease was diagnosed and successfully treated.

*Conclusions:* Sirolimus uses after kidney transplantation may lead to lung complications, especially BOOP, and the prompt diagnosis would allow earlier treatment.

## 1. Introduction

Sirolimus is a macrolide immunosuppressive lactone isolated from *Streptomyces hygroscopicus * which is known as proliferation signal inhibitors. Sirolimus was being used as an alternative to calcineurin inhibitors in organ transplant recipients with low nephrotoxicity since 1999 (1). Now it is also used to prevent restenosis in patient with coronary artery stents, polycystic kidney disease, hamartomatous diseases and treat metastatic cancer. Some side effects have been reported following administration of sirolimus including thrombocytopenia, hyperlipidemia, acne, bone marrow suppression, edema, proteinuria and aphthous ulcers (2). Pulmonary complications of sirolimus are uncommon, but if it occurs, can be life threatening. In this regard, one of the diseases that is discussed more recently, is bronchiolitis obliterans organizing pneumonia (BOOP), which is a condition that may be caused by respiratory infectious pneumonias, other infectious agents, specific medications, connective tissue and immunologic diseases, organ transplantation, and certain occupational and environmental exposures and in some cases also can be idiopathic (3,4). Here we reported a case of BOOP associated with sirolimus in patients with history of kidney transplantation. To our knowledge, this case is the first report from Iran.

## 2. Case presentation

**Figure 1:**
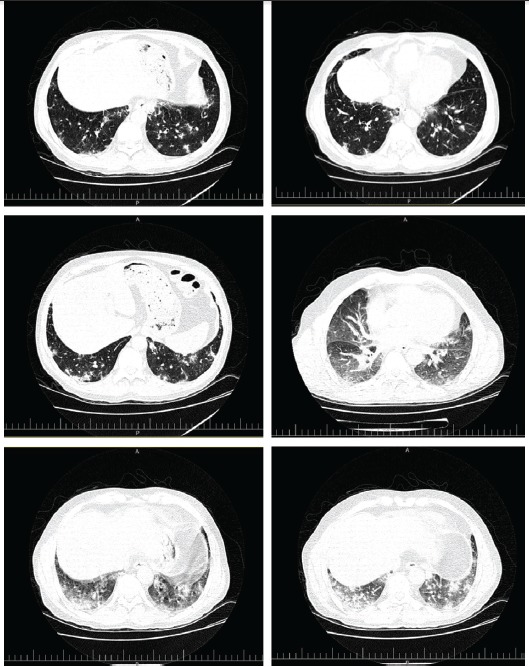
Computed Tomography studies of patients with sirolimus-associated pneumonitis. Mediastinum vascular swelling, reticular densities, septal thickness, bronchial wall thickness, mild bronchiectasis, patchy and nodular alveolar densities on the inferior lobe of both lungs.

**Figure 2:**
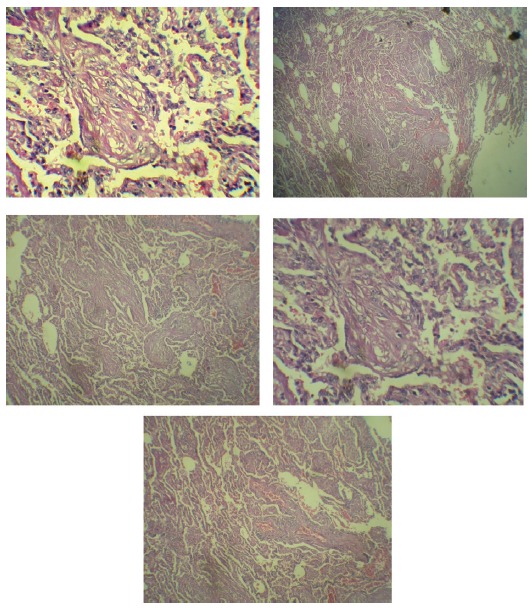
Microscopic views of histologic specimen. Patchy pattern of nodules of granulation tissue in and around small airways consists of polypoid plugs showing fibroblast in an edematous stroma in the lumen of bronchioles into lumens of adjacent alveolar ducts and alveoli. There are moderate of lymphocyte plasma cells in some bronchioles and interstitial inflammation. An intra-alveolar collection of foamy macrophages is also existed.

We report a case of 49-years-old male who received a kidney from a live unrelated donor since 7 years ago. During these seven years multiple low grade fevers was being appeared every year that healed without a diagnosis. On 29 May 2013, he presented with 39 °C fever associated with dysuria, oliguria, right lower quadrant abdominal pain, anorexia, cough and dyspnea. The patient had also history of diabetes mellitus, hypertension and gout. Blood pressure measured 130/90 mmHg, pulse rate 96 per minute, and respiratory rate 18 per minute. Laboratory tests were as follow: BUN= 19 mg/dl, Cr= 2 mg/dl, Na= 133 mEq/l, K=4.5 mEq/l, FBS=130 mg/dl, ESR= 50 mm/hour, CRP= 44.1 mg/dl, WBC= 5.8 ×10^6^/µL, RBC= 4.02 × 10^6^/µL, Hg= 11.3 g/dl, hematocrit= 33.6%, MCV 83.58 fl, and platelet= 171 × 10^6^/µL. In sonography investigation, his kidney graft was 123×55 mm with normal parenchymal echo. The test for cytomegalovirus (CMV Ag-pp65) was negative and enterobacter growth was seen in the blood culture. In high resolution computed tomography (HRCT), an increase in heart size, mediastinum vascular swelling, reticular densities, septal thickness, bronchial wall thickness, mild bronchiectasis, patchy and nodular alveolar densities on the inferior lobe of both lungs were seen. Spiral CT scan showed micro-consolidation patchy lesions on the inferior lobe of both lungs (Figure 1 ). Bronchoscopy showed hyper-vascularity in the trachea, and in broncho-alveolar lavage (BAL) growth of non-A, non-D streptococci were detected with no growth of acid fast bacilli or mycobacterium tuberculosis. After 17 days of antibiotic and antifungal therapy, his fever did not corrupt. Spirometry results, showed a decreased FEV1 to 75%. On June 23th, inferior lobe wedge resection of the left lung was performed. In macroscopic pathologic examinations, fragments of lung tissue measuring 5×2×2 cm and gray brown and soft tissue in serial cross sections were observed.**Moreover*, * microscopic evaluations conducted on issue removed during a procedure (Figure 2 ). Finally, the diagnosis of BOOP was reported with no evidence of malignancy. Sirolimus was withdrawn and cyclosporine was started and dosage of prednisolone was increased. His fever was discontinued and general condition as well as pulmonary symptoms were gradually improved.

## 3. Discussion

Pneumonitis developed in our patient following long-term use of sirolimus. Clinical and pathologic findings were indicative of sirolimus-induced pulmonary toxicity. Sirolimus is a macrolide immunosuppressive agent that was first approved for use in renal transplantation in 1999. Sirolimus-induced pneumonitis was first reported in 2000 in renal transplant patients (1,5). Moreover, it is reported with other organ transplants, including liver, heart, islet cells and lung.

The exact etiology, pathogenesis, and the optimal treatment for post-transplantation BOOP is unclear. BOOP has several etiologies, however, sirolimus-induced pneumonitis is mentioned in fewer studies (6). Nevertheless, its incidence seems to be increased in renal transplant patients taking sirolimus in recent years from 5% to 15%. However, in a recent randomized controlled trials conducted on 800 patients treated with sirolimus no cases of BOOP were reported (7).

This complication was also reported associated with some other proliferation signal inhibitors such as everolimus and temsirolimus (8). It is believe that the probable mechanism of action of this drug is through T-lymphocyte activation by interfering with interlukein-2 signal transduction (9).

The most frequent reported side effects of drug are hyperlipidemia, thrombocytopenia and increased liver function enzymes. Besides, some cases of pulmonary involvement are reported too. Lung toxicity followed by sirolimus may be presented as a BOOP, interstitial pneumonitis, non-cardiogenic pulmonary edema and alveolar hemorrhage. Base on the information of literatures, symptoms occur after 1 to 51 months after initiation of sirolimus therapy (10). Here in our case, the patient had self-removing mild periodic fever during last seven years, which cause was not detected by clinicians. BOOP is a respiratory condition that is regarded as a response to acute and sub-acute lung injury and describe with presence of granulation tissue within the bronchial lumen.

BOOP has many etiologies. Totally, it is divided into two entities; idiopathic and secondary. Causes of secondary BOOP may result from inhalation of toxic substances, connective tissue diseases, drugs, and infections by bacteria, viruses (such as chlamydia pneumonia, Coxiella Burnetii, Adenovirus), parasite and fungal infections (Plasmodium vivax, Cryptococcus neoformans), immunologic disorders, malignancies, radiation therapy, alcoholic cirrhosis, chronic thyroiditis, bone marrow/lung transplantation and inflammatory bowel disease (4,7). The present case had none of above triggers or disorders which confirmed through exact clinic and para-clinic evaluations.

Risk factors of this type of pneumonia include older age, male gender, serum level/dose of immunosuppression, disturbance in kidney function which greatly consistent with our patient.

Most reported cases of sirolimus-induced lung toxicity were associated with high dose of drug.

High doses are used for cancer diseases. In this patient, pulmonary involvement may sometimes be detected in thoracic imaging in asymptomatic patients. Presented case has not the history of malignancy (11,12).

BOOP usually presents as a flu-like illness. The typical presentations are nonspecific systemic symptoms (e.g. fevers, chills, night sweats, fatigue and weight loss) and respiratory symptoms (e.g. dyspnea and cough) along with pulmonary changes which are visible in thoracic imagines. Clinical suspicious is important in diagnosis of BOOP (13). After the clinical features and chest radiographic findings were in favor of BOOP, it is necessary confirm the diagnosis with histopathologic finding. Also, BAL has been shown to be effective in all suspected cases to rule out other disorders such as malignancy and infection (14). However, three main imaging patterns suggest BOOP in chest X ray (15,16): 1) patchy alveolar and diffuse interstitial infiltrates, usually bilaterally; 2) multiple foci of consolidation or solitary nodular appearing lesions; and 3) appearance of small linear or crescent-shaped densities surrounding the ground glass area of attenuation. In addition, HRCT shows ground glass attenuation with a sub-plural and peri-bronchial distribution (13). Although both clinical manifestations and radiological findings may suggest BOOP, histological findings are required to confirm the diagnosis. Definitive histological results of BOOP are as subsequent: 1) diffuse distal airway inflammation and fibromyxoid and presence of polypoid plugs; 2) large interstitial and alveolar infiltrates that contain mononuclear cells and “foamy” macrophages; and 3) patchy involvement of pulmonary parenchyma with preservation of background lung architecture (13,16).

Corticosteroids are the main treatment for BOOP. The recommended dosing of prednisone is 0.75 to 1 mg/kg per day for 3 months, then 40 mg/d for 3 months, and finally tapering to 20 mg/d or 20 mg every other day for 6 months, for a total treatment time of approximately 1 year (11,12). The majority of patients recover within days or weeks such as our patient. However, in a few patients, the disease may persist. Relapse occurs in approximately 30% of patients just after withdrawal of treatment. Some other drugs such as erythromycin, inhaled triamcinolone, azathioprine, cyclosporine and cyclophosphamide may be useful for treatment of BOOP (17,18).

## 4. Conclusions

Sirolimus uses after kidney transplantation may lead to lung complications, especially BOOP, and the prompt diagnosis would allow earlier treatment.

## Conflict of interests

None of the contributing authors have any conflict of interest, including specific financial interests or relationships and affiliations relevant to the subject matter or materials discussed in the manuscript.

## Authors’ contributions

All authors wrote the paper equally.

## Funding/Support

None.
